# ShapeShop: Towards Understanding Deep Learning Representations via Interactive Experimentation

**DOI:** 10.1145/3027063.3053103

**Published:** 2017-05

**Authors:** Fred Hohman, Nathan Hodas, Duen Horng Chau

**Affiliations:** College of Computing, Georgia Institute of Technology Atlanta, GA 30332, USA; Data Sciences & Analytics, Pacific Northwest National Laboratory, Richland, WA 99354, USA; College of Computing, Georgia Institute of Technology, Atlanta, GA 30332, USA

**Keywords:** Interactive visualization, model exploration, learning semantics, 1.2.m [Artificial Intelligence]: Miscellaneous

## Abstract

Deep learning is the driving force behind many recent technologies; however, deep neural networks are often viewed as “black-boxes” due to their internal complexity that is hard to understand. Little research focuses on helping people explore and understand the relationship between a user's data and the learned representations in deep learning models. We present our ongoing work, ShapeShop, an interactive system for visualizing and understanding what semantics a neural network model has learned. Built using standard web technologies, ShapeShop allows users to experiment with and compare deep learning models to help explore the robustness of image classifiers.

## Introduction

Commonly, researchers and practitioners working in deep learning build a model iteratively, as they search a hyper-parameter space to find the best set of parameters to construct the best model possible. This exploratory process takes a nontrivial amount of time and is often guided by the model builder's intuition and experience. This is especially true in deep learning, where models can perform rather differently depending on the data and specific neural network being considered [7]. To expedite this process and build better models, researchers want to be able to understand what a model has learned, to help decide what to try next.

**Figure 1:**
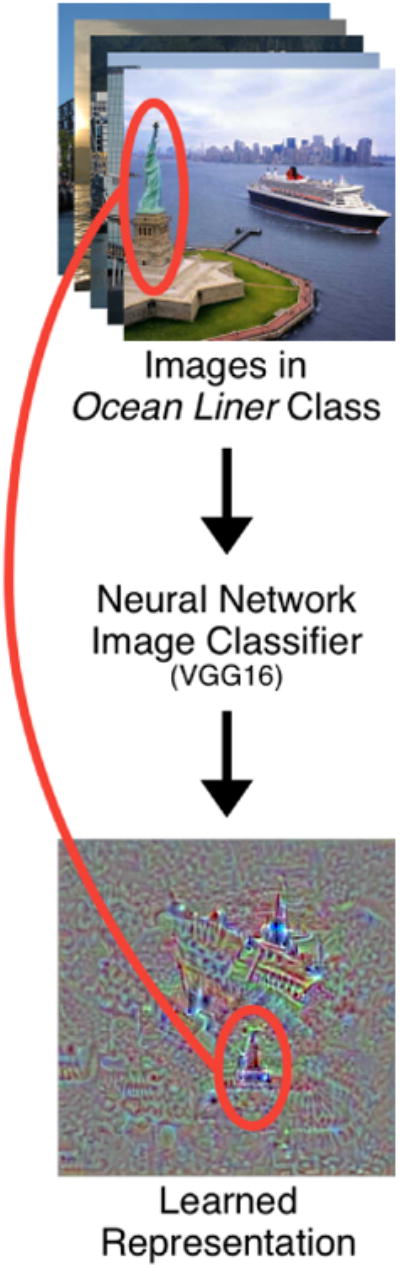
Visualizing the *ocean liner* class learned by the VGG16 [8] network (bottom) showing the network possibly learning other semantically less related image objects to the ocean liner (e.g., Statue of Liberty, circled in red).

Interactive visualization techniques have been used to probe machine learning models to understand what they have learned [1, 6]; in deep learning, new techniques have been developed to specifically inspect neural network models [10, 11]. Specifically for neural network image classifiers, a visualization technique called class activation maximization [5] shows what a classifier has learned for each class (e.g., Fig. 1, bottom). While keeping the layer weights of a neural network fixed after the training phase, one maximizes a class's output by initializing it with an initial image and optimizing the image while using the model loss function as the score, activating the specific neurons that correspond to that class [2]. Back-propagation is performed until the model is certain the numerically generated image belongs to the target class. The bottom image in Fig. 1 shows an example of this procedure being applied to the popular VGG16 convolutional neural network image classifier [8] showing the *ocean liner* class. The hope is that the resulting image should represent the prototypical class, i.e., what the model understands that specific class to be, since all of its relevant neurons have been activated. This technique can be used to produce images on a wide range of networks, for example, MNIST digit classifiers [2], and has even been used to produce realistic images [4, 5].

However, research has only started looking into how to support people in exploring and interpreting what a deep learning model has learned [9, 3]. Using a deep learning model as a black-box can be detrimental — if it performs poorly, users may not know why, leading to flawed decisions. For example, consider Fig. 1, showing class activation maximization for the ocean liner class on the VGG16 [8] network. Notice the boat with *ocean liner-like* features; however, also notice the unidentifiable object circled in red (possibly the Statue of Liberty or a distant building). The network has learned that this object, which has no semantic connection to the ocean liner, is somehow representative of the ocean liner. This kind of incorrectly learned representation illustrates that using model accuracy alone may be insufficient as a measure of model robustness. By understanding the learned representations, users can confidently deploy a model and trust the decisions it makes.

We are addressing this important research problem of deep learning interpretation by developing the foundational tools and techniques to help users explore the relationship between image data used in classification tasks and the learned representations. Currently our work supports a small image dataset consisting of shapes; we are extending it for more benchmark datasets (e.g., CIFAR10, ImageNet). Our ongoing work presents the following contributions:

1. The Shape Workshop (ShapeShop): an interactive visualization for users to explore, experiment with, and visualize the relationship between a training dataset and the learned model space, in real time.
2. Preliminary experimental results showing what semantics various models are learning in simple shapes, by using ShapeShop to compare multiple models.

## ShapeShop

**Figure 2:**
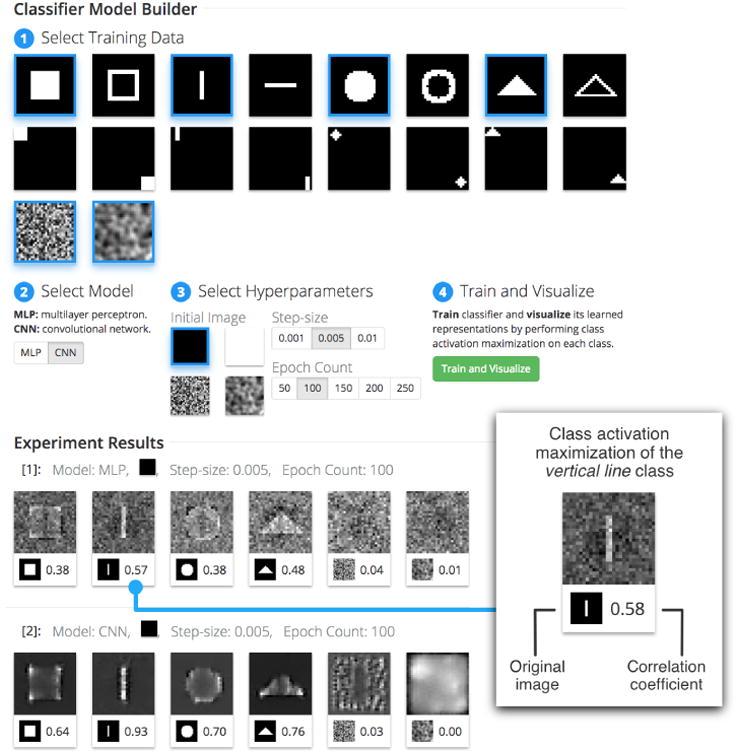
The ShapeShop system user interface is divided into two main sections. The Model Builder (top) contains the training data, model, and hyperparameter selection options where a user follows enumerated steps, concluding with the system building and training an *N*-image classifier, where each training image selected corresponds to one class. In the Experiment Results section (bottom), each time the “Train and Visualize” button is clicked a new set of results appears including the class activation maximization of each class.

ShapeShop is an interactive system (see Fig. 2) for visualizing what semantics a neural network has learned from data consisting of simple shapes in images (Fig. 2.1). It enables interactive model experimentation, where a user can explore and compare various image classifiers and their learned spaces. It does so by training an *N*-image neural network classifier where the user visually selects which *N* classes to include during model training. A handful of model hyperparameters can also be selected prior to training, such as the model choice (a multilayer perceptron or a convolutional neural network, Fig. 2.2), the initial image used in the image generation process (an all black image, an all white image, noise, and blurred noise), the gradient ascent step-size, and the number of epochs used in training (Fig. 2.3), however the system is easily extendable to include any model hyperparameter desired.

ShapeShop uses the class activation maximization visualization technique to produce *N* images, each corresponding to one class. The system presents all *N* resulting images and correlation coefficients back to the user for visual inspection and comparison. This process then repeats, where a user can select different images to train on, produce more visualizations from new models, and compare to the previous results. This process provides feedback to the user to assist with model building, giving a deeper understanding of what space the model is learning. Note that every model trained in ShapeShop is trained to near perfect accuracy (>99%) and reports a low loss score, reinforcing that models can be trained to similar accuracy but learn completely different spaces. Our focus is on fundamentally understanding what the neural network models are capable of learning, not the quality or photo-realness of the produced images; therefore we resist from realism-boosting gradient update heuristics, as seen in other research [4].

### System Design & Implementation

For the back-end, we use the Keras (https://keras.io) Python package, a wrapper for deep learning libraries such as Ten-sorFlow and Theano, for model building, training, and image generation. To achieve close to real-time results, we limit our images to be small in size, and for each training image clicked, we include ten of the image with random noise added to produce unique training data. We have designed ShapeShop with portability in mind; ShapeShop also runs on machines with different computational capabilities (e.g., laptops, GPU-equipped machines) and scale to larger datasets. The system contains two primary models to choose from: a multilayer perceptron containing two fully connected dense layers of width 128 followed by a dense softmax classification layer, and a simple convolutional neural network containing two convolutional layers, a max pooling layer, and ending with the same softmax layer for classification. However, the system is agnostic to the model, so any other model can be swapped out.

For the front-end, we use modern web technologies such as D3 to display data selection and image generation results. ShapeShop enumerates the steps for using the system. These are 1: *Select Training Data*, 2: *Select Model*, 3: *Select Hyperparameters*, and 4: *Train and Visualize*. Once the data and hyperparameters have been selected and the “Train and Visualize” button is clicked, the image generation process begins. The results appear in a row at the bottom of the view and include one image and correlation coefficient corresponding to each training image selected before.

The image represents the prototypical class, i.e., what semantics the neural network has learned. Since an image is produced for each class, the user is able to visually inspect the result and qualitatively compare it to the original black and white binary image. The correlation coefficient provides a more quantitative comparison to the original binary image used in training. ShapeShop uses the absolute value of the Pearson correlation coefficient. Therefore, the coefficient ranges from 0 to 1, where numbers closer to 0 indicate low to no correlation found between the produced image and original, and numbers closer to 1 indicate high correlation between the produced image and the original.

## Preliminary Results

We now present two scenarios where ShapeShop helps a user explore how deep learning models learn different representations given diverse data and model architectures.

### Understanding the Effect of Diverse Data

A user wants to explore the effect of adding more diverse data to a model to see how the model's representation changes. Maybe augmenting a model with more diverse classes could build a more accurate model with representations that are more human recognizable, or perhaps it may be the exact opposite — more data could mean a less accurate model and less recognizable representations.

Our user starts with two specific classes in Fig. 2: the centered vertical and horizontal line classes. Using ShapeShop, we build, train, and visualize a binary classifier by choosing the black initial input image in the hyperparameter selection section and keeping all other hyperparameters with default values. In the results, Fig. 3.1, we see two model representation images: the left for the vertical line class, and the right for the horizontal line class. Our user sees that the model is interestingly classifying one class (e.g., vertical line) by the *absence* (dark horizontal line) of their other. This surprises our user — a line should be defined by its straight path, and not the absence of other paths.

Since the user wants the model to learn human recognizable accurate representations of both line classes, the user adds two more classes: solid and hollow circles. Our user expects that the model will now focus on more than just vertical and horizontal directions, such as the curves found in the circle classes. Keeping all other parameters fixed, the user builds, trains, and visualizes the model (shown in Fig. 3.2). The user notices the dark lines in each of the line classes are now diminished, indicating that adding the two circle classes helped the model learn more accurate semantics of the lines. As a result, the line correlation coefficients increase to 0.52 and 0.53. Following this observation, the user wants to continue to improve the model's semantic representations, so the user user adds two more classes to include: the noise and the blurred noise classes. This indeed helps; the model now focuses more on the image features now that noise has been included (Fig. 3.3), further improving the correlation coefficients to 0.59 and 0.57.

Through ShapeShop, our user gains insight into how having diverse data can help the model learn more human recognizable representations, going beyond typical validation metrics such accuracy and loss.

### Comparing Different Neural Network Architectures

The user now wants to compare the multilayer perceptron (MLP) network to the convolutional neural network (CNN) architectures. CNNs are built and tuned to seek out spatial correlations in images by using filters to detect gradients, edges, curves, and other conceptual semantics, while MLPs are not as robust for detecting such features. Using ShapeShop, the user selects four shapes and both noise classes to add to the training data and visualizes two models: an MLP and a CNN (keeping all other hyperparameters fixed, Fig. 2). Notice that the user's expectations are confirmed: while the MLP (Fig. 2.[1]) captures some semantic qualities of each shape, the CNN (Fig. 2.[2]) better shows what parts in each image are distinctive from the rest of the set. For example, notice the corners of the square being highlighted to distinguish its class from the circle class, where corners do not exist.

## Ongoing Work and Conclusion

### Highlighting Semantic Objects

**Figure 4:**
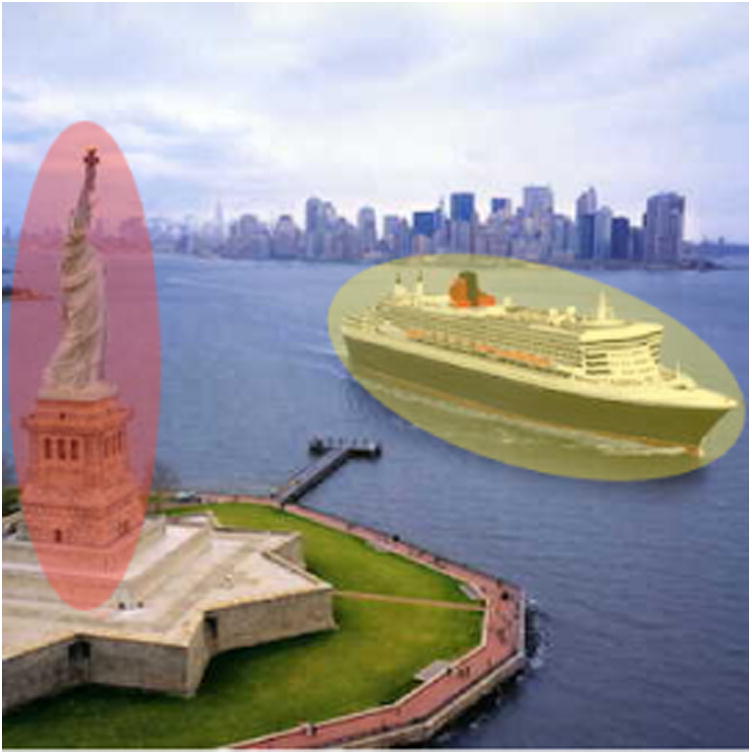
An example data case when a user highlights semantic object features in the training dataset. The user-highlighted yellow region is the relevant semantic part of the image desired to be classified, most commonly the image's class (in this example, the *ocean liner*) and the user-drawn red region denotes that this semantic object is not part of the desired class.

Visualizing class representations of a neural network helps in understanding what a model has both learned and not learned, providing insight on how to iterate the model in the future. As we saw in Fig. 1, the *ocean liner* representation contained an object that did not belong to the class, possibly showing that our model thinks some object (e.g., the Statue of Liberty) is part of the boat. We plan to explore how incorporating human feedback could help address this problem via a semantic highlighting feature that we think could help improve a model's learned representations and even shorten model training time. As seen in Fig. 4, a user is presented with a data case from the *ocean liner* class and highlights the boat with a yellow (positive) region and the Statue of Liberty with a red (negative) region, providing additional semantically meaningful constraints to the model during training. We believe this technique could improve model accuracy when a user's dataset is small and each data case is majorly influential during the training process.

### Freeform Class Drawing

Currently ShapeShop is a web-based interactive system for visualizing model representations from a selectable set of training data consisting simple shapes in images. Planned work includes the addition of a new UI component in the *Select Training Data* section where a blank canvas allows a user to draw their own image class (or derive from an existing class). Alongside the predefined set of images currently available, providing this free form drawing tool could allow users to create more complex geometries to further experiment with neural network classifiers. For example, creating compositions of simple semantics could test whether a model improves given its components, e.g., does a house class (triangle on top, square on bottom) improve in the presence of square and triangle classes.

### Planned Evaluation

Besides evaluating ShapeShop's usability, we plan to recruit deep learning researchers to evaluate the utility our planned features and system as a whole. We plan to test how the semantic highlighting feature may improve model accuracy, shorten the model training time, and produce more semantically accurate learned representations. Also, we plan to experiment with the freeform drawing tool to explore how the presence of simple compositional semantics may affect the recognition of more complex geometries.

### Conclusion

Through ShapeShop, we have taken an important first step in helping users visualize and better understand deep learning representations via interactive experimentation. We plan to extend ShapeShop's capabilities to support interactive semantic highlighting and freeform class drawing, and we believe effective combination of visual and quantitative techniques will ultimately help users gain insights into and illuminate black-box deep learning models.

## Figures and Tables

**Figure 3:**
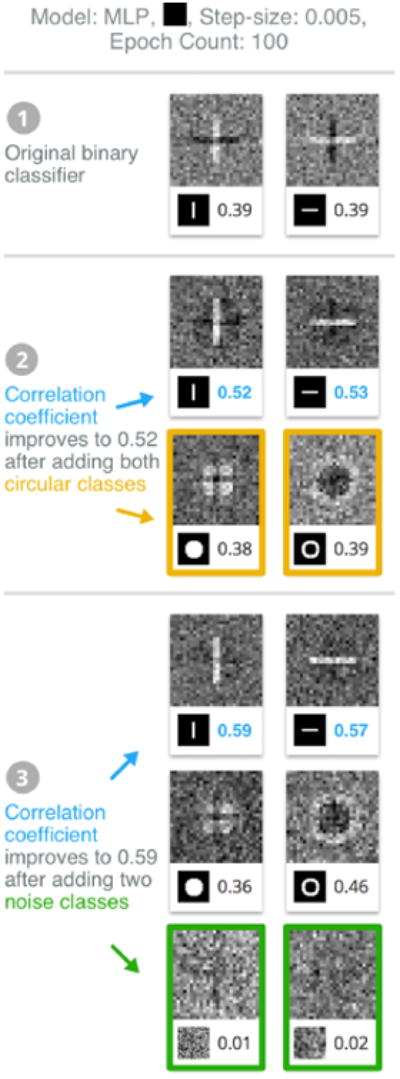
Through ShapeShop, our user understands how data diversity improves a classifier's semantic robustness. ❶ The user starts with two line classes, whose quality incrementally improves ❷ by adding circle classes and ❸ noise classes.
